# Face and Word Recognition Can Be Selectively Affected by Brain Injury or Developmental Disorders

**DOI:** 10.3389/fpsyg.2017.01547

**Published:** 2017-09-06

**Authors:** Ro J. Robotham, Randi Starrfelt

**Affiliations:** Department of Psychology, University of Copenhagen Copenhagen, Denmark

**Keywords:** visual perception, prosopagnosia, alexia, face recognition, word recognition, reading, hemispheric specialisation

## Abstract

Face and word recognition have traditionally been thought to rely on highly specialised and relatively independent cognitive processes. Some of the strongest evidence for this has come from patients with seemingly category-specific visual perceptual deficits such as pure prosopagnosia, a selective face recognition deficit, and pure alexia, a selective word recognition deficit. Together, the patterns of impaired reading with preserved face recognition and impaired face recognition with preserved reading constitute a double dissociation. The existence of these selective deficits has been questioned over the past decade. It has been suggested that studies describing patients with these pure deficits have failed to measure the supposedly preserved functions using sensitive enough measures, and that if tested using sensitive measurements, all patients with deficits in one visual category would also have deficits in the other. The implications of this would be immense, with most textbooks in cognitive neuropsychology requiring drastic revisions. In order to evaluate the evidence for dissociations, we review studies that specifically investigate whether face or word recognition can be selectively affected by acquired brain injury or developmental disorders. We only include studies published since 2004, as comprehensive reviews of earlier studies are available. Most of the studies assess the supposedly preserved functions using sensitive measurements. We found convincing evidence that reading can be preserved in acquired and developmental prosopagnosia and also evidence (though weaker) that face recognition can be preserved in acquired or developmental dyslexia, suggesting that face and word recognition are at least in part supported by independent processes.

## Introduction

Face and word recognition have traditionally been thought to rely on highly specialised and relatively independent cognitive processes. Some of the strongest evidence for this has come from neuropsychological case studies. There are many examples of patients suffering from pure prosopagnosia, a selective deficit in face recognition, and patients suffering from pure alexia, a selective reading deficit (for comprehensive reviews see Farah, 1990, 2004). Together, the patterns of face recognition deficits with preserved reading, and reading deficits with preserved face recognition constitute a double dissociation, which is current textbook knowledge (Gazzaniga et al., 2013).

The hemispheric lateralisation of face and word recognition has been considered additional evidence for their functional independence. Pure prosopagnosia occurs after bilateral or right hemisphere damage, whereas pure alexia arises after left hemisphere damage (Farah, 1991; Leff et al., 2006; Barton, 2008b; Starrfelt and Shallice, 2014). Also, functional imaging studies have shown that a region in the left fusiform gyrus, the visual word form area (VWFA) is more responsive to words (Puce et al., 1996; Cohen et al., 2000) whereas a part of the right fusiform gyrus, the Fusiform Face Area (FFA) is more responsive to faces (Kanwisher et al., 1997). This aligns well with ERP-studies: The N170 component for faces has a larger amplitude in the right hemisphere while the N170 for words has a larger amplitude in the left hemisphere (Bentin et al., 1996; Schendan et al., 1998).

Imaging studies also show, however, that the lateralisation of activation for faces and words is far from complete, as both categories lead to bilateral activation with variable degrees of asymmetry, suggesting that they might rely on neural networks that are highly overlapping (Dien, 2009; Nestor et al., 2013).

Findings from neuropsychological studies have also challenged the idea of functional independence. There are a few patients on record with prosopagnosia following lesions restricted to the left hemisphere and patients with alexia following damage restricted to the right hemisphere (Davous and Boller, 1994; Mattson et al., 2000; Barton, 2008a). A greater challenge comes from a study reporting face recognition deficits in alexia patients with left hemisphere damage and word recognition deficits in prosopagnosics with right hemisphere damage (Behrmann and Plaut, 2014). This finding, combined with imaging and modelling results, led the authors to propose a distributed model of visual recognition: the many-to-many hypothesis (MTMH: Behrmann and Plaut, 2013). According to the MTMH, the cortical networks supporting face and word recognition are not specialised for specific categories but instead involved in processing a whole range of visual categories. Face and word recognition are supported by common and overlapping networks that are bilaterally distributed, rather than by independent modules. The contributions of the left and right hemisphere are, however, differently weighted for the two categories (Behrmann and Plaut, 2013). A key prediction of the hypothesis that has been described explicitly by the authors is that face recognition problems should be present in all patients with pure alexia and reading deficits should accompany all cases with pure prosopagnosia (Plaut and Behrmann, 2013). In other words, the authors question whether a dissociation between face and word recognition exists. This has led to an increase in studies specifically investigating whether face and word recognition can be selectively impaired.

Dissociations are considered as a key tool in neuropsychology for identifying independent mental processes. Double dissociations are more powerful than single dissociations as they cannot be explained by differences in task difficulty. Although double dissociations have some methodological limitations (e.g., Dunn and Kirsner, 2003), most researchers still consider them the strongest inferential tool available in neuropsychology for establishing whether two processes are separate (Shallice, 1988; Coltheart, 2001).

Associations refer to patterns where a patient’s performance is impaired in two tasks after brain injury (Coltheart, 2001). Association does not necessarily imply, however, that the two functions rely on common and overlapping processes. For example, it is possible that the functions rely on independent processes located spatially close in the brain, so that both were affected by the same lesion. Another possibility is that the two functions rely on some common and some independent processes. Abnormal performance in face recognition and word recognition following injury could for example be due to blurry vision. It does not exclude the possibility that faces and words, at a higher level, rely on independent processes.

Findings of dissociations between face and word recognition would provide strong evidence that faces and words are not supported by fully distributed processes but instead at least in part by independent processes. Also, such dissociations would constitute evidence against one of the original key predictions of the MTMH (Susilo and Duchaine, 2013).

## Methods

Farah (1990, 2004) has provided comprehensive reviews of studies of visual agnosia for faces and words up to 2004. In this paper we review 15 studies published since 2004 specifically investigating whether face or word recognition can be selectively affected by acquired brain injury or developmental disorders.

## Results

We start by describing studies that selected patients according to their symptoms and then describe studies that selected participants according to the location of their lesions (see Supplementary Table 1 for more details on each study).

### Face Recognition in Acquired Alexia and Developmental Dyslexia

Two studies report face recognition deficits in patients with pure alexia or in patients with unilateral lesions in the left posterior fusiform gyrus (pFG), when assessed with a range of sensitive tests. Testing face and word recognition in four patients with pure alexia (Behrmann and Plaut, 2014) showed that the patients with pure alexia showed mild but significant deficits on simultaneous face discrimination tasks and had abnormal face inversion effects. A drawback of this study is that the results are only based on comparisons between very small groups and that individual test scores are not reported (but the significance level of individual scores are reported!).

A study of face processing in 19 patients with lesions in the left ventral occipito-temporal cortex and/or who had an abnormally high word-length effect (Roberts et al., 2015) reports similar findings. Patients were slower and less accurate than controls on a face naming task and slower (not less accurate) on a face-to-name matching task and half of them were also impaired when compared individually to the control group. Interestingly, longer RTs on a simultaneous face discrimination task were associated with more severe reading deficits (higher word length effect).

In contrast, two single case studies have reported preserved face recognition in patients with acquired alexia. An epilepsy patient was shown to have unimpaired face processing following resection of a word responsive area in the occipito-temporal cortex which resulted in alexia (Gaillard et al., 2006). However, while the patient’s impaired function, reading, was assessed with various sensitive tests (RTs and Acc.), the supposedly preserved function, face processing was assessed using only accuracy in a quite crude test, the 25 item Warrington face recognition test. An interesting finding in this patient was that fMRI activation patterns for faces that were restricted to the RH before surgery did not change following surgery, while the “selective” activation elicited by visual words disappeared. Another study with similar findings (Turkeltaub et al., 2014) describes a patient who, following a selective lesion in the inferior left occipito-temporal cortex (corresponding to the VWFA), shows an abnormal word length. The patient has a normal performance on a subtest of the Philadelphia Face Perception Battery, which is a relatively sensitive, accuracy based task.

A few studies have investigated face recognition in developmental dyslexia, showing mixed results. One study report that a group of 18 participants with developmental dyslexia were not significantly slower or less accurate than a group of controls on a face naming task (Smith-Spark and Moore, 2009). The study did, however, show that there were larger age of acquisition effects in the control group compared to the dyslexia group, which the authors suggested could be related to attentional or executive dysfunctions in the dyslexia group. In contrast, in a study investigating face and complex object recognition in subjects with developmental dyslexia (Sigurdardottir et al., 2015) dyslexics were on a group level reported to perform significantly worse than controls on two face recognition tests. According to the authors, the face recognition deficits seen in dyslexics in this study do not seem to be caused by a deficit in holistic processing, which many consider a core deficit in prosopagnosic patients.

### Reading in Acquired and Developmental Prosopagnosia

In addition to reporting impaired face processing in alexia, Behrmann and Plaut (2014) also reported three patients with prosopagnosia who showed abnormally long RTs and word-length effects on reading tasks. This study has, however, been criticised (Hills et al., 2015) for including a prosopagnosia patient that had previously been described in the literature as having integrative agnosia (Behrmann and Kimchi, 2003).

Other studies have provided evidence that reading can be preserved in acquired prosopagnosia. Five patients with acquired prosopagnosia were tested on seven sensitive tests of word recognition and four patients performed normally (Acc. and RT) on all tasks when compared individually to the control group (Susilo et al., 2015).

Another study investigated word processing in two patients with prosopagnosia following stroke in the right hemisphere, one patient with prosopagnosia following herpes simplex encephalitis, as well as one patient with pure alexia following stroke in the left hemisphere (Barton et al., 2010). The participants had to sort words by word identity or by style (font or handwriting). The two prosopagnosic patients were impaired in sorting words according to script style (Acc. and/or RTs) but performed normally when sorting for word identity, whereas the alexic patient had the opposite pattern. The herpes simplex patient performed normally on both tasks. The results suggest that while patients with prosopagnosia can have unimpaired reading, other aspects of word processing, such as style, might be affected in these patients.

Yet another study testing six prosopagnosia patients with unilateral right lesions and five with bilateral lesions (Hills et al., 2015) found that none of the patients with unilateral lesions showed abnormal word-length effects or RTs in word-naming. Patients also carried out the sorting task mentioned above (Barton et al., 2010), and again patients were as fast as controls in sorting the words by identity but many were slower than controls in sorting the words according to handwriting or font style.

A few single case studies have also described intact reading in acquired prosopagnosia (see Supplementary Table 1 for details). One subject with face recognition problems following traumatic brain injury had a very fast reading rate of 364 words per minute when reading text (Bukach et al., 2006). Another patient with severe face processing deficits was shown to have normal accuracy on the word part of the Warrington Recognition Memory Test (Riddoch et al., 2008).

Reading can also be unaffected in developmental prosopagnosia. In one study, 10 developmental prosopagnosics performed well within the normal range on four sensitive tests of letter, word, and text reading, and a dissociation was demonstrated statistically between impaired face and preserved word recognition (Starrfelt et al., 2016).

Similarly, Rubino et al. (2016) assessed reading in ten developmental prosopagnosics using a word-naming and word-sorting task (according to content or style, cf. Barton et al., 2010). At a group level, there was no difference between the prosopagnosic and controls group regarding errors, mean RTs and word-length effects. And at the individual level none of the prospagnosics had elevated word-length effects. In contrast to subjects with acquired prosopagnosia assessed with the same task in a previous study, only one subject was impaired in sorting by font (Hills et al., 2015).

### Large Studies Using Anatomy-Based Inclusion Criteria

Two studies included patients on the basis of lesion location rather than symptomatology. A large patient study investigated 31 patients with Posterior Cerebral Artery (PCA) stroke with sensitive experimental face, house, object, and word processing tests (Martinaud et al., 2012). Face processing deficits were observed after right and after left hemisphere damage. Word processing deficits were, however, only found in patients with left hemisphere lesions. Interestingly, although six patients had deficits for a single category (house, phone, word, or face), only one of these, a patient with a house processing deficit, had a truly selective deficit according to the stringent Revised Standardized Difference Test (Crawford and Garthwaite, 2005). Another study investigating visual perceptual abilities in 31 patients with unilateral, subacute stroke in regions supplied by the PCA found that many patients with left hemisphere lesions had face recognition deficits and that many patients with right hemisphere lesions also had reading deficits (Gerlach et al., 2014).

### Methodological Considerations: Levels of Processing

All the studies included compare performances in word and face processing, and discuss whether these functions rely on common or selective mechanisms. In many of these studies, however, the tests used measure different levels of processing for faces than for words (see Supplementary Table 1). While some experiments tap processing at a perceptual level that requires very little semantic knowledge, others require identification of specific stimuli. Overall, there are three broad groups of tests (see **Table 1**): (1) Perceptual tasks like simultaneous matching that can be performed without the subject having to store a cohesive representation of the stimulus. (2) Recognition tasks that require subjects to build and store short-term or longer-term representations, such as the Cambridge Face Memory Test (Duchaine and Nakayama, 2006). And (3) Identification tasks, like famous face tests or reading out loud, which require associating the perceived stimulus with stored semantic and/or phonological representations. Some studies also use key behavioural effects like the word length effects and the face inversion effects as proxies for reading and face processing functions.

**Table 1:** Examples of commonly used tests sorted according to level of processing.

	Word processing	Face processing
Perception	• Sorting words according to content or style (Barton et al., 2010)	• Cambridge face perception test (Duchaine et al., 2007)
		• Benton test of face recognition (Benton et al., 1994)
		• Simultaneous discrimination task (e.g., Behrmann and Plaut, 2014)
Recognition	• Warrington recognition tests for words (Warrington, 1984)	• Warrington recognition tests for faces (Warrington, 1984)
	• Lexical decision (e.g., Gaillard et al., 2006).	• Cambridge face memory test (Duchaine and Nakayama, 2006)
		• Delayed matching task (e.g., Riddoch et al., 2008)
		• Familiarity judgement test (e.g., Bukach et al., 2006)
Identification	• Naming words (WLE) (e.g., Starrfelt et al., 2016)	• Famous faces tasks: naming or matching (e.g., Roberts et al., 2015)


If the aim is to draw conclusions about the extent to which face and word processing rely on common mechanisms, then matching tasks to measure the same level(s) of processing is important.

## Discussion

The studies described show mixed results. Deficits in reading and face recognition sometimes co-occur. But this does not necessarily imply that the two functions rely on shared processes only. For example, if face and word processing were supported by independent processes closely located in the brain, a single lesion affecting both would lead to co-occurring deficits. Also, face and word processing must rely on some common processes, that if damaged, lead to abnormal performances in both types of tasks. For example, blurred vision could affect performance on a wide range of visual tasks and language deficits could affect performance on face and word tasks that require naming.

More importantly, our review shows that there is convincing evidence that dissociations between the two functions can be found, suggesting that face and word recognition, at least in part, rely on independent processes. As pointed out by Susilo and Duchaine (2013), this contradicts one of the original key predictions of the many-to-many hypothesis, that reading and face recognition deficits should always co-occur (Behrmann and Plaut, 2013).

Interestingly, at this point, the evidence for dissociation is stronger in one direction (preserved reading in prosopagnosia) that in the other. This might be for trivial reasons not related to the main question, but it is also possible that such dissociations (preserved face recognition in alexia or dyslexia) are rarer or less reliable. The most obvious explanation for a single dissociation, however, is that one task (face recognition) is simply more demanding than the other (reading) (Shallice, 1988). This seems counter intuitive here as face recognition is innate while reading is learned. Recent evidence suggests, however, that there is a systematic relationship between the two functions cognitively and cerebrally and that learning to read might directly affect the cerebral substrate for face processing. Before learning to read, ventral occipito-temporal cortex responds bilaterally to faces, but increases in reading ability in children and preliterate adults are associated with a reduced left hemisphere response to faces (Dehaene et al., 2010; Cantlon et al., 2011). Studies using divided visual field paradigms and ERP measurements in children and adults have shown similar results (Mercure et al., 2009; Dundas et al., 2013, 2014).

The neuronal recycling hypothesis (Dehaene and Cohen, 2007) explicitly describes how learning to read might affect the neural substrates of face processing. Reading recycles pre-existing brain systems that are genetically defined for other uses, specifically in the left ventral occipito-temporal (vOT). The vOT shows a preference for high-resolution foveal shapes, and is well-adapted to extracting line configurations consistent with requirements for word recognition (Dehaene and Cohen, 2007). The left lateralisation allows shorter connections to language areas [indeed the connectivity of the VWFA in pre-reading children predicts its location following reading acquisition (Saygin et al., 2016)]. Because of these constraints regarding localisation, word processing competes with face processing for cortical space in the left vOT, and as one becomes more proficient in reading and the left vOT is tuned for word recognition, face processing becomes more right lateralised (Dehaene and Cohen, 2011). This hypothesis could potentially account for why reading can be preserved in prosopagnosia but that face recognition problems are likely to be seen in people with dyslexia (see Ventura, 2014, for a recent review of how reading acquisition and face recognition could be related).

In conclusion, while there is convincing evidence that reading can be preserved in acquired and developmental prosopagnosia, evidence that face recognition can be preserved in acquired or developmental dyslexia is somewhat weaker. Taken together the results suggest that face and word recognition are at least in part supported by independent processes. More detailed investigations of face recognition in dyslexia are needed to determine whether face processing can be preserved when reading is impaired, and thus whether there is a reliable double dissociation between face and word recognition.

## Author Contributions

RR selected relevant articles for the review and wrote the first draft of the manuscript. RS provided a revised version of the manuscript. The manuscript was then finished in close collaboration between the authors.

## Conflict of Interest Statement

The authors declare that the research was conducted in the absence of any commercial or financial relationships that could be construed as a potential conflict of interest.
